# Generational and gendered perceptions of weightlifting: a cross-sectional survey

**DOI:** 10.3389/fspor.2026.1829361

**Published:** 2026-07-08

**Authors:** Lauren Butler, Ciarra Cayo, Amanda Thomas, Jeffrey Fernandez, Mark D. Rossi, David Capote, Haley Bushman, Fedline Sylvestre, Gisselle Gonzalez, Sophia Ulman

**Affiliations:** 1 Physical Therapy, Florida International University, Miami, FL, United States; 2 Department of Rehabilitation, Nicklaus Children's Hospital, Miami, FL, United States; 3 Movement Science Lab, Scottish Rite for Children, Frisco, TX, United States; 4 Department of Orthopaedic Surgery, University of Texas Southwestern, Dallas, TX, United States

**Keywords:** gender, strength training, weight training perceptions, weightlifting beliefs, youth

## Abstract

**Introduction:**

This study aimed to describe perceptions of weightlifting across generations, and to examine differences toward women and youth cross-generationally.

**Methods:**

295 Participants (40.1 ± 16.3 [18-78] years, 62.4% female) completed an electronic survey capturing demographics, weightlifting history, and weightlifting perceptions. An adapted version of the Multidimensional Outcome Expectations for Exercise Scale (MOEES) assessed weightlifting perceptions using a 5-point Likert scale (1- Strongly Disagree to 5-Strongly Agree). Participants completed the MOEES for themselves and for four randomized case scenarios (8-year-old child, 15-year-old teen, 30-year-old woman and a 30-year-old man). Participants were grouped by birth year (Gen Z, Millennials, Gen X, Baby Boomers, and the Silent Generation). Kruskal-Wallis tests assessed differences in MOEES scores by generational group. Pairwise comparisons were performed with Bonferroni-adjusted p-values (*p* < 0.05).

**Results:**

Baby Boomers reported less favorable perceptions than Gen Z and Millennials for their own participation in weightlifting (Baby Boomers vs. Gen Z, *p* = 0.002; Baby Boomers vs. Millennials, p<0.001) and for men's participation (Baby Boomers vs. Gen Z, *p* < 0.001; Baby Boomers vs. Millennials (*p* = 0.001). For women, greater differences were observed, with Baby Boomers reporting less favorable perceptions than Gen Z (*p* < 0.001) and Millennials (*p* < 0.001). Less consistent trends were observed for the youth scenarios. For the teen scenario, Baby Boomers reported significantly lower perceptions compared to Gen Z (*p* < 0.001) and Millennials (*p* = 0.019). For the child, Baby Boomers differed from Gen Z and Millennials with lower scores (*p* = 0.005-0.034). Similarly, Gen X indicated significantly lower perceptions compared to Millennials (*p* = 0.024).

**Discussion:**

The results highlight less favorable perceptions of weightlifting among older generations, with declining expectations across multiple domains for each older generation. These findings underscore the need for targeted education on the safety and efficacy of weightlifting, particularly for individuals from older generations.

## 1 Introduction

Weightlifting, a subset of resistance training, is a form of exercise that uses external resistance to strengthen muscles and improve physical performance (1). Decades of evidence support its role in overall health and wellness through its ability to enhance musculoskeletal health, bone density, cardiovascular function and psychological well-being (2, 3). In addition, weightlifting also supports injury prevention, improves body mechanics, and promotes greater independence across the lifespan (4).

These benefits are particularly important for women, who have lower baseline muscle strength compared to men, and who are at an increased risk for osteoporosis, particularly after menopause (5, 6). Similarly, improvements in body image, self-esteem and overall self-worth have been reported in youth who engage in weightlifting (7, 8). In terms of rehabilitation, weightlifting is recognized as a critical tool for restoring individuals' prior level of function, improving neuromuscular control, and preventing further functional decline (2). Despite this, participation in weightlifting remains lower than recommended in several populations, most notably among women, older adults, and youth (9, 10).

Participation in weightlifting has undergone drastic changes over the decades and across generations. Historically, women have demonstrated low engagement, often due to limited education about the benefits of resistance training and societal discouragement (11, 12). Cultural perspectives suggested that women feared becoming “too muscular,” and time spent away from domestic duties was viewed negatively (11). Qualitative studies confirm that women from older generations often lacked awareness of benefits and even received advice discouraging them from lifting heavy objects. Instead, they were steered more toward aerobic-based exercise such as dance, walking, and light household activity, rather than weightlifting (11). Among youth, concerns of potential injuries, growth plate damage, and stunted growth from weightlifting were common, raising fears about the possible negative effects of children lifting weights (13).

In contrast, today's young adult populations reflect the largest growth in women's participation in weightlifting, influenced by the shifting of cultural attitudes, increased access to gyms, and the increase in social media fitness culture (14, 15). Gender and generational differences have also been observed in other areas, including sports participation and health behaviors. For example, the percentage of girls participating in sports has increased from 24.2% in 1973 to 42.9% in 2018 (16). Similarly, the prevalence of adults, not meeting physical activity guidelines declined roughly 10% from 1997 to 2018 (16). Yet, even within this generation, women continue to encounter gym intimidation, fear of judgment, and stereotypes about muscularity and masculinity (17, 18). Although weightlifting is more socially accepted for both men and women, the participation rates still reflect male dominance (19). While resistance training is now widely accepted and recognized as a safe and effective intervention to increase muscular strength in youth, overall muscular strength in youth has declined (13, 20–22). Reduced free play opportunities, increased sedentary behaviors, and the changing youth sports landscape are thought to be contributing factors (13). In response, the American Academy of Pediatrics now recommends that children engage in resistance training, which includes weightlifting (13).

Although the benefits of weightlifting are well established, participation rates remain below the recommendations set by the American College of Sports Medicine, which states strength training should be done at least twice weekly (23). Women consistently fall short of these guidelines, with adherence rates as low as 20% among college-aged women (11, 17). Similar trends have been observed in youth populations, with a national study using Youth Risk Behavioral Survey data reporting a decline in resistance training participation from 55.6% in 2011 to 49.5% in 2019, with boys being more likely to meet guidelines than girls (24). This disconnect raises a key question: if the benefits of weightlifting are widely understood and recognized, why do so few women and children engage in it? While there are individual barriers such as time, access, and physical limitations (15, 25), studies rarely explore how perceptions of weightlifting are shaped across generations or how these insights differ by gender and age. It has been theorized that individuals from the same generation tend to have similar values, behaviors, and perceptions which have been shaped by shared life experiences (26–28). For the purpose of this study, we will describe those characteristics as generational culture. Current research tends to isolate specific age groups, limiting our ability to understand intergenerational trends and the cultural collaboration of these beliefs.

To address this gap, this study seeks to describe perceptions of weightlifting across generations and examines differences toward women and youth, cross-generationally. Given historical gender stereotypes and negative cultural perceptions related to safety for youth, this study hypothesizes that individuals from older generations would hold more negative perceptions of weightlifting in general, and particularly of women and youth who participate in weightlifting.

## 2 Methods

### 2.1 Study design

A cross-sectional survey study design was used. A convenience sample of adult participants aged 18 years and older were invited to participate in an anonymous electronic survey collected in Qualtrics (Provo, UT, USA) (29). The survey link was distributed via electronic mailing lists and social media, with potential participants encouraged to share the survey with their peers, facilitating snowball sampling. Adult participants 18 years of age and older were included for participation. Those younger than 18 years were excluded. This study was approved by the Florida International University Institutional Review Board IRB # IRB-25-0024. All participants provided informed consent prior to participation, in accordance with the Declaration of Helsinki.

### 2.2 Procedures

The survey included three sections: 1) demographic information, 2) weightlifting participation, and 3) perceptions of weightlifting. Specifically, the demographic section captured participant age, sex, race, ethnicity, and birth year. Birth year was used to subsequently categorize participants into one of five generational groups: Generation Z (Gen Z: 1997–2006), Generation Y or Millennials (1981–1996), Generation X (Gen X: 1965–1980), Baby Boomers (1946–1964), and Silent Generation (1928–1945). The weightlifting participation section asked if participants had ever engaged in weightlifting during their lifetime (past participation) and if they currently participate in weightlifting (current participation), using a dichotomous yes/no response.

Finally, the Multidimensional Outcome Expectations for Exercise Scale (MOEES) was adapted to assess weightlifting perceptions. The MOEES is a 15-item questionnaire that assesses the beliefs and expectations of an individual regarding the benefits of regular exercise across three domains: physical, social, and self-evaluative (30). The physical domain reflects beliefs about positive and negative physical experiences resulting from engagement in physical activity. The social domain reflects beliefs about how physical activity impacts opportunities for socialization and social approval. Finally, the self-evaluative domain reflects beliefs related to feelings of satisfaction and self-worth from physical activity engagement (30). Participants are asked to rate their agreement on a 5-point Likert scale (1- strongly disagree to 5-strongly agree) with statements of the possible benefits of exercises. The MOEES has demonstrated acceptable reliability and validity in various populations (30–32).

In this study, the word “exercise” was replaced with “weightlifting” in the MOEES to assess outcome expectations of weightlifting. For example, the original statement of “Exercise will increase my muscle strength,” was modified to read “weightlifting will increase my muscle strength.” All modified MOEES statements can be found in Table 1. Participants first completed the modified MOEES relating to their own participation in weightlifting (SELF). Next, they were provided with four scenarios; a 30-year-old man (MAN), a 30-year-old woman (WOMAN), a 15-year-old teen (TEEN), and an eight-year-old child (CHILD), and were asked to complete the modified MOEES for the individuals in each scenario. Specifically, they were asked to rate their agreement for the possible benefits of weightlifting for individuals stated in each scenario. The scenarios were presented in a randomized order using the Qualtrics randomization function, with randomization intended to balance the order of presentation across participants. The survey took approximately 10 min to complete.

**Table 1:** Modified MOEES statements.

MOEES Domain	Items of SELF
Physical Outcome Expectations	Weightlifting will improve my ability to perform daily activities Weightlifting will strengthen my bones Weightlifting will aid in weight control
Social Outcome Expectations	Weightlifting will improve my social standing Weightlifting will make me more at ease with people
Self-evaluative Outcome Expectations	Weightlifting will help manage stress Weightlifting will improve my mood

Items were adapted from the original MOEES to assess weightlifting as the target behavior. Items were repeated for MAN, WOMEN, CHILD, and TEEN by modifying the reference (example: Weightlifting will improve “a woman's” ability to perform daily activities).

MOEES, Multidimensional outcomes expectations for exercise scale.

Total scores and domain scores (physical, social, self-evaluative) were calculated for each scenario (Self, Man, Women, Teen, Child) by summing the responses to the corresponding statements. The total score ranges from 15 to 75 with a higher score indicating stronger positive expectations about the outcomes of weightlifting, or more specifically, that weightlifting will produce beneficial outcomes across physical, social, and psychological dimensions. Physical domain scores range from 6 to 30, and higher scores indicate stronger expectations that weightlifting will improve physical health, fitness, or appearance. The social domain score can range from 4 to 20 and greater scores reflect stronger beliefs that weightlifting will enhance social interactions, approval, or connectedness. Lastly, the self-evaluative domain score ranges from 5 to 25 with higher scores indicating greater expectations that weightlifting will improve self-esteem, confidence, or personal satisfaction.

### 2.3 Statistical analysis

Descriptive statistics (means, standard deviations, medians, and interquartile ranges) were calculated for all outcome variables, including total and domain (physical, social, and self-evaluative) scores of the modified MOEES across each scenario (SELF, MAN, WOMAN, TEEN, and CHILD). Internal consistency reliability of the modified MOEES was assessed using Cronbach's alpha (33, 34). Alpha was calculated separately for each of the five survey scenarios.

Given the data did not meet assumptions of normality, nonparametric tests were employed. Independent-samples Kruskal–Wallis tests were used to examine generational differences total and domain-specific MOEES scores for each scenario. When significant results were identified, pairwise comparisons were conducted with Bonferroni correction applied to adjust for multiple testing. Effect sizes (*r*) were calculated for all pairwise comparisons to quantify the magnitude of observed differences (35, 36). Statistical significance was set at *p* < 0.05. All statistical analyses were conducted using IBM SPSS Statistics (version 24.0; IBM Corp., Armonk, NY, USA).

## 3 Results

Two-hundred and ninety-seven participants [40.34 ± 16.67 (18–87) years, 62.0% female] completed the survey and were included for analysis (Table 2). The majority of participants were Gen Z or Millennials, followed by Baby Boomers, Gen X, and the Silent Generation. Generational trends in weightlifting experience and participation are reported in Table 2. Overall, Millennials had the greatest number of participants reporting experience in weightlifting and current participation in weightlifting.

**Table 2:** Demographics, weightlifting experience, and current participation across generations.

Measure	Gen Z (1997−2006)	Millennials (1981–1996)	Gen X (1965–1980)	Baby Boomers (1946–1964)	Silent Generation (1933–1945)
N, %	98, 33.0	93, 31.3	50, 16.8	54, 18.2	2, 0.7
Sex (% Female)	72, 73.5	55, 59.1	26, 52.0	31, 57.4	0, 0.0
Race (% White)	75, 77.3	84, 90.3	40, 81.6	51, 94.4	2, 100.0
Ethnicity (% Hispanic)	44, 44.9	44, 47.3	25, 50.0	12, 22.2	1, 50.0
Past Experience (% Yes)	89, 90.8	92, 98.9	46, 92.0	46, 85.2	1, 50.0
Current Participation (% Yes)	64, 71.9	69, 75.0	29, 63.0	34, 73.9	0, 0.0

Values are presented as mean ± standard deviation for continuous measures and median [interquartile range] for ordinal measures.

Cronbach's alpha values for the modified MOEES ranged from *α* = 0.84–0.87 across the five scenarios, indicating good internal consistency. Scenario-specific alpha values were: SELF, *α* = 0.84; MAN, *α* = 0.85; WOMAN, *α* = 0.85; TEEN, *α* = 0.86; and CHILD, *α* = 0.87. Generational differences in total and domain-specific MOEES scores are presented below for each scenario. Notably, no statistically significant differences with the Silent Generation were identified due to a small sample size of two. However, average scores are still included in Table 3 to reflect a generational trend.

**Table 3:** Total and domain-specific MOEES scores for each scenario by generation.

Generation	Gen Z	Millennials	Gen X	Baby Boomers	Silent Generation
SELF					
Physical	26.3 ± 4.4	27.3 ± 3.5	26.1 ± 5.0	24.6 ± 5.6^b^	21.0 ± 1.4
Social	13.3 ± 3.9	13.0 ± 3.9	12.6 ± 4.0	11.4 ± 3.4	13.0 ± 2.8
Self-Evaluative	21.5 ± 3.8	22.1 ± 3.2	21.1 ± 4.1	19.0 ± 4.2^a^^,^^b^^,^^c^	18.0 ± 2.8
Total	61.1 ± 10.7	62.3 ± 8.5	59.8 ± 11.0	54.9 ± 10.6^a^^,^^b^	52.0 ± 4.2
MAN					
Physical	27.0 ± 3.5	27.0 ± 3.9	26.9 ± 3.4	24.5 ± 5.6^a^^,^^b^	24.5 ± 2.1
Social	16.2 ± 3.4	15.3 ± 3.5	14.5 ± 4.1	13.0 ± 3.8^a^^,^^b^	14.0 ± 2.8
Self-Evaluative	22.2 ± 3.1	21.9 ± 3.5	21.5 ± 3.5	19.5 ± 4.3^a^^,^^b^	18.5 ± 2.1
Total	65.4 ± 8.8	64.3 ± 9.0	62.9 ± 9.7	57.0 ± 11.5^a^^,^^b^	57.0 ± 7.1
WOMAN					
Physical	27.3 ± 3.2	27.1 ± 3.8	26.3 ± 4.7	24.2 ± 4.3^a^^,^^b^^,^^c^	22.0 ± 0.0
Social	15.5 ± 3.6	14.0 ± 3.6^a^	14.0 ± 4.4	12.5 ± 3.0^a^	13.5 ± 2.1
Self-Evaluative	22.3 ± 2.8	21.9 ± 3.6	21.3 ± 4.4	19.2 ± 3.8^a^^,^^b^^,^^c^	18.5 ± 2.1
Total	65.1 ± 8.4	63.0 ± 8.9	61.6 ± 11.5	55.9 ± 9.2^a^^,^^b^^,^^c^	54.0 ± 4.2
TEEN					
Physical	26.0 ± 4.0	26.1 ± 3.6	25.6 ± 3.9	23.1 ± 4.9^a^^,^^b^	21.5 ± 2.1
Social	16.2 ± 3.3	15.1 ± 3.2	14.8 ± 3.3	13.9 ± 3.7^a^	15.0 ± 1.4
Self-Evaluative	21.6 ± 3.1	21.4 ± 3.1	21.1 ± 3.3	19.5 ± 4.1^a^	18.0 ± 2.8
Total	63.8 ± 9.3	62.6 ± 8.6	61.4 ± 9.1	56.5 ± 11.4^a^^,^^b^	54.5 ± 2.1
CHILD					
Physical	21.7 ± 6.7	22.6 ± 6.0	18.6 ± 6.9^b^	18.0 ± 6.2^a^^,^^b^	18.5 ± 4.9
Social	12.4 ± 4.6	12.2 ± 4.2	10.3 ± 4.1	10.5 ± 3.6	12.0 ± 5.7
Self-Evaluative	17.6 ± 5.3	18.3 ± 5.4	15.7 ± 5.9	15.2 ± 5.0^b^	16.0 ± 4.2
Total	51.7 ± 15.7	53.0 ± 14.4	44.6 ± 15.5^b^	43.6 ± 14.3^a^^,^^b^	46.5 ± 4.9

Values are reported as means ± standard deviations.

Significant pairwise group comparisons indicated with a superscript if different from;

a Gen Z

b Millennials

c Gen X

d Baby Boomers

### 3.1 Self-assessment

**Figure 1:**
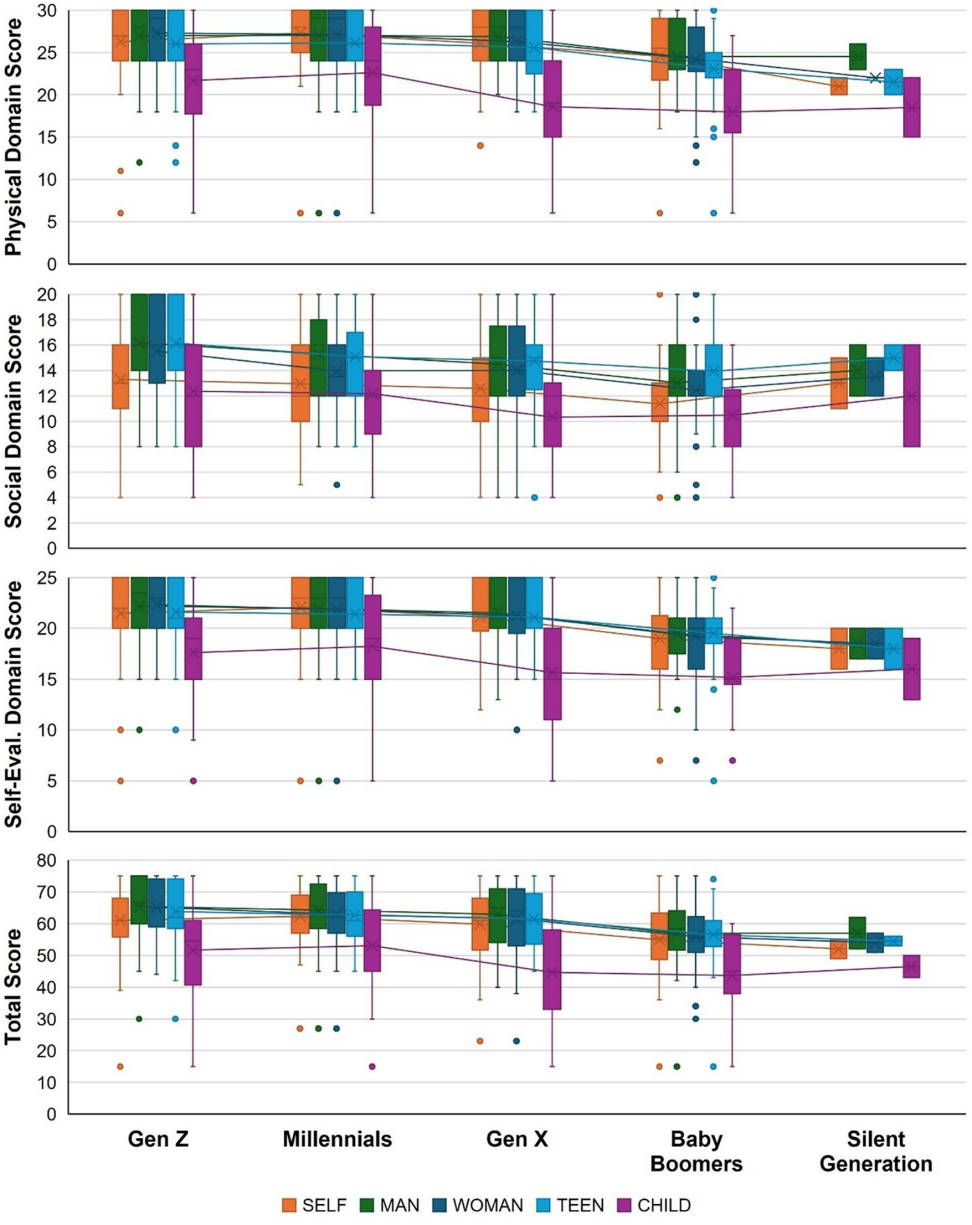
Box and whisker plots illustrate the physical, social, and self-evaluative domain scores, as well as the total score of the MOEES across generations for each scenario. Boxes represent the interquartile range, the horizontal line within each box indicates the median, and the “×” symbol denotes the mean value. Whiskers extend to 1.5 times the interquartile range, and circles represent outliers.

Weightlifting perceptions of the participants' own practices as well as across the various scenarios differed between generations (Table 3, Figure 1). When asked about their own expectations with weightlifting, Baby Boomers consistently indicated lower expectations than younger generations. Specifically, Baby Boomers reported significantly lower expectations than Millennials in the physical domain (*p* = 0.012, *r* = −0.28), Gen Z, Millennials, and Gen X in the self-evaluative domain (*p* < 0.001–0.013, *r* = −0.31 - −0.41), and Gen Z and Millennials overall (*p* < 0.001−0.002, *r* = −0.31 - −0.36).

### 3.2 MAN scenario

For the scenario in which a 30-year-old MAN is weightlifting, similar generational differences were found (Table 3, Figure 1). Baby Boomers consistently indicated significantly lower expectations across all three domains (physical: *p* *=* 0.006−0.011, *r* = −0.28 - −0.29; social: *p* < 0.001−0.010, *r* = −0.27 - −0.40; self-evaluative: *p* = 0.001−0.002, *r* = −0.32 - −0.35) and overall (total: *p* < 0.001−0.001, *r* = −0.34 - −0.40), specifically compared to Gen Z and Millennials. While not statistically significant, Gen X also reported higher expectations relative to Bay Boomers, but generally did not reach the level of expectations indicated by Gen Z and Millennials.

### 3.3 WOMAN scenario

Similar to the MAN scenario, expectations reported for the 30-year-old WOMAN scenario decreased with each older generation (Table 3, Figure 1). Baby Boomers consistently indicated significantly lower expectations for the physical (*p* < 0.001−0.026, *r* = −0.28 - −0.39) and self-evaluative (*p* < 0.001−0.016, *r* = −0.30 - −0.41) domains, as well as overall (total: *p* < 0.001−0.013, *r* = −0.29 - −0.46), compared to Gen Z, Millennials, and Gen X. Additionally, both Millennials (*p* = 0.035, *r* = −0.22) and Baby Boomers (*p* < 0.001, *r* = −0.40) reported significantly lower expectations for the social domain compared to Gen Z. However, although not statistically significant, Millennials indicated higher expectations for the social domain compared to Baby Boomers.

### 3.4 TEEN scenario

While expectations for the 15-year-old teen followed a similar declining trend across generations, fewer significant group differences were found (Table 3, Figure 1). Baby Boomers reported significantly lower expectations compared to Gen Z for all domain scores (physical: *p* = 0.001, *r* = −0.34; social: *p* = 0.002, *r* = −0.31; self-evaluative: *p* = 0.043, *r* = −0.25) and overall (total: *p* < 0.001, *r* = −0.35). Baby Boomers also significantly differed from Millennials specifically for the physical domain (*p* = 0.002, *r* = −0.33) and total (*p* = 0.019, *r* = −0.27) scores.

### 3.5 CHILD scenario

Trends in the expectations for the benefits of weightlifting slightly differ for the eight-year-old child scenario (Table 3, Figure 1). Unlike prior scenarios, MOEES scores do not consistently decrease across older generations. Baby Boomers similarly differed from Gen Z and Millennials with lower scores for the physical domain (*p* < 0.001−0.007, *r* = −0.28 - −0.36) and overall (total: *p* = 0.005−0.034, *r* = −0.24 - −0.30), and a lower self-evaluative domain score compared to Millennials (*p* = 0.015, *r* = −0.27). Additionally, similar to Baby Boomers, Gen X indicated significantly lower expectations compared to Millennials for the physical domain (*p* = 0.007, *r* = −0.29) and overall (total: *p* = 0.024, *r* = −0.26) scores. However, while not statistically significant, Gen Z reported lower expectations, on average, for nearly all scores compared to Millennials, which reported the highest expectations across generations. The social domain score is the only exception to this trend in which Gen X and Millennials reported nearly the same level of expectations.

## 4 Discussion

The purpose of this study was to describe perceptions of weightlifting across generations and to examine differences toward women and youth, cross-generationally. Our hypothesis, that individuals from older generations would hold more negative perceptions of weightlifting in general, and particularly toward women and youth who participate in weightlifting was supported. For the SELF, MAN, WOMAN, and TEEN scenario, expectations for weightlifting decreased with each older generation, with the most consistent trends observed for the SELF, MAN, and WOMAN scenarios. Expectations for the CHILD scenario followed a less consistent declining trend.

When asked about their own expectations of weightlifting, older generations indicated lower expectations across the physical and self-evaluative domain, and overall. However, no differences were observed for the social domain. While not specifically explored in this study, the authors postulate that this pattern may be influenced by long-standing societal beliefs that older adults are frail or at higher risk for injury, leading to the perception that resistance training or higher-intensity physical activity is unsafe for aging populations. These assumptions continue despite the evidence demonstrating that properly prescribed resistance training is safe and effective for older adults and is associated with improvements in strength, bone density, functional capacity, and cardiometabolic health (37).

Given the observed differences in the self-evaluative domain, the authors theorize that psychological factors may further contribute to lower self-expectations for weightlifting among older generations It is well known that aging is often accompanied by increased sensitivity to pain (38), concerns about symptom exacerbation (39), and the accumulation of chronic health conditions (40). These factors may influence how older adults perceive their capacity for exercise and subsequently may lower self-expectations for engaging in weightlifting. While no differences in expectations were observed in the social domain, it is possible that peer influence may still play a role in participation. Social norms and peer participation have been identified as strong predictors of physical activity behavior across the lifespan (41). Furthermore, research demonstrates that resistance training participation among older adults increases when it is normalized within community and social contexts (42).

From a clinical and public health perspective, these findings highlight the importance of reframing resistance training as not only a tool for functional independence, disease management, and psychological well-being for aging adults, but also as a tool to build muscle mass and enhance appearance. Targeted education that addresses these benefits may help shift expectations and increase participation across older generations, ultimately supporting healthier aging habits.

When presented with the 30-year-old man and women scenarios, lower expectations were reported across all three domains (physical, self-evaluative, and social), and overall, for each older generation and each scenario. One possible explanation for these findings is that expectations for weightlifting during early to mid-adulthood may be affected by increasing life demands that intensify with age. While this was not specifically examined in the current study, it is known that time constraints, perceived lack of energy, and the belief that one has aged beyond their physical “prime” are among the most reported barriers to exercise participation in adults (43, 44). Thus, the authors speculate that as occupational responsibilities, caregiving demands, and psychological stress increase, structured exercise, particularly resistance training, which is often perceived as time-intensive, may be deprioritized, contributing to declining expectations across older generations.

Greater differences in expectations were observed for the women scenario compared to the man scenario across all generations. The authors propose that this may also be explained by gender-specific sociocultural factors. For example, adults in their 30s and beyond are more likely to assume caregiving roles for children and aging family members, which has been associated with reduced leisure-time physical activity, particularly among women (45). While pregnancy status was not measured in the current study, pregnancy and the postpartum period may further shape perceptions, as misconceptions may portray women as fragile or in need of excessive activity restriction, despite evidence that appropriately prescribed resistance training during pregnancy is safe and beneficial (46). Men, in contrast, may experience lower expectations for weightlifting due to perceived role demands related to work and financial provision, which could reduce the time and priority allocated to exercise and other health-promoting behaviors. With these considerations, the authors posit that lower expectations for both men and women lifting weights may not solely be driven by physical capability, but by evolving social roles, time limitations, and enduring gender norms—factors that vary across generations and continue to shape perceptions of weightlifting participation. Future studies should explore this further.

Finally, for the two youth scenarios (TEEN and CHILD), less consistent trends were observed across generations. Older generations expressed less favorable expectations toward children and teens' participation in weightlifting across multiple domains. However, for the child scenario, the most notable differences were observed in the physical domain. It is the author's opinion that these findings may be related to misconceptions about potential negative consequences of youth-weightlifting. In the past, concerns related to growth plate damage or stunted growth from children and teens' engagement in weightlifting were commonly circulated. However, these concerns have been discredited, with no adverse effects reported in the literature (47). In response to these concerns, several professional organizations have published position statements regarding the safety and efficacy of youth resistance training, reflecting a cultural shift in youth weightlifting acceptance. This shift may help explain the generational differences observed in this study. Furthermore, this may be particularly relevant for children, given that the most consistent significant differences were observed in the physical domain for the child scenario.

Interestingly, no differences were observed between generations for the social domain of the child scenario. Overall, responses generally reflect neutral social expectations for a child participating in weightlifting. This may potentially highlight a lack of awareness of the social benefits of weightlifting for children across all generations. While there is evidence for the positive benefits of weightlifting on self-esteem, resistance training confidence, and self-worth in youth, limited works discuss the social impacts of weightlifting. Given the role of social factors in exercise participation, future work should aim to specifically examine the social benefits of youth weightlifting, such as improving a sense of belonging and building positive peer relationships.

Finally, Millennials reported the highest expectations for the child scenario across all domains compared to the other generations. While not explored in the current study, the authors speculate that this may be related to the age of Millennials (ranging from 29 to 44 years) which typically aligns with the “childbearing” years. It is plausible that participants with children may be more familiar with recommendations made by health care professionals regarding children's physical activity, which may include weightlifting. It is also possible that participants' prior experience with their own child's participation in weightlifting may have influenced their responses. As such, this should be explored in future work.

### 4.1 Limitations

This study has several limitations that need to be considered. First, and most importantly, while the modified MOEES demonstrated good internal consistency, additional psychometric properties were not examined. Thus, results should be interpreted with caution. Next, while the MOEES was used to assess expectations of weightlifting for each scenario, we were unable to gather more in-depth information related to why participants held these expectations. As such, future studies should use qualitative methodology to gain a deeper understanding of the drivers of these perceptions. The child and teen scenarios used in this study were also intentionally written as gender neutral. However, not assigning gender to these scenarios may limit our understanding of gender-specific perceptions in those age groups and this should be explored in future research. Additionally, the snowball sampling methodology may have introduced sampling bias which may reduce the generalizability of findings. Similarly, the sample was predominantly white, female, and composed of participants with weightlifting experience which further limits the generalizability. This study also did not control for participants' weightlifting experience which may have influenced study findings given that experience may influence perceptions. Likewise, race and ethnicity may have influenced participants' perceptions and should be examined in future research. Importantly, the weightlifting participation questions asked participants dichotomously about their past and current participation in weightlifting. This did not allow for the quantification of weightlifting frequency among participants, which may have influenced perceptions. Finally, given that responses were self-reported, it is also possible that participants' perceptions of socially acceptable answers may have influenced their own answer choices, which may have impacted the accuracy of study findings.

## 5 Conclusion

The results of this study highlight less favorable perceptions of weightlifting among individuals from older generations, with consistently declining expectations observed across multiple domains for each older generation. However, expectations for the child and teen scenarios showed less consistent trends. Overall, these findings underscore the need for targeted education related to the safety and efficacy of weightlifting, particularly for individuals from older generations, to promote weightlifting participation.

## Data Availability

The datasets presented in this article are not readily available because data is not publicly available. Requests to access the datasets should be directed to Lbutler@fiu.edu.
